# Vaccines, media and politics: A corpus-assisted discourse study of press representations of the safety and efficacy of COVID-19 vaccines

**DOI:** 10.1371/journal.pone.0279500

**Published:** 2022-12-30

**Authors:** Ming Liu, Ruinan Zhao, Cindy Sing Bik Ngai

**Affiliations:** 1 Department of Chinese and Bilingual Studies, The Hong Kong Polytechnic University, Kowloon, Hong Kong, People’s Republic of China; 2 Faculty of Humanities, The Hong Kong Polytechnic University, Kowloon, Hong Kong, People’s Republic of China; Georgia State University, UNITED STATES

## Abstract

This study gives a corpus-assisted discourse study of the representations of the safety and efficacy of COVID-19 vaccines in three representative newspapers from the US, Hong Kong, and the Chinese mainland: *New York Times* (NYT), *South China Morning Post* (SCMP), and *China Daily* (CD). The primary purpose is to explicate the dynamics between vaccines, media, and politics. Combining the theories and methods of critical discourse analysis and corpus linguistics, this study has revealed their preferential ways of constructing the safety and efficacy of COVID-19 vaccines at different levels of discourse. The safety and efficacy of COVID-19 vaccines thus serve as an important ideological battlefield for newspapers from different origins to advance their respective national or regional interests and shape understanding of different COVID-19 vaccines in the international arena.

## 1. Introduction

Since the World Health Organization (WHO) declared the novel coronavirus (COVID-19) outbreak as a global pandemic on 11 March 2020, COVID-19 vaccines have been regarded as one of the most effective means to stop the spread of the pandemic [1]. Although vaccines have been available for COVID-19, there is still widespread distrust and scepticism towards them in different parts of the world [2, 3]. Media have always been regarded as an important means of spreading vaccine (mis)information and shaping public understanding of vaccines [4–6]. It plays an important role in keeping vaccine scares and scepticisms alive, because individuals tend to be exposed to different types of information via media and cannot discern fact from misinformation or speculation [7]. This has given rise to numerous studies on media representations of vaccines in the last two decades [8–10]. For example, Wang [8] examined the legitimization strategies for a vaccine scandal in China’s official media. Mohr and Frederiksen [10] also gave a critical discourse analysis of the representation of HPV vaccine controversy in Danish media. They have illuminated not only the particular ways of vaccine representations but also the socio-political factors behind these representations.

Previous studies have also verified that public concerns over vaccine safety and effectiveness are among the main causes for vaccine hesitancy [11, 12]. A recent study on the effects of media frames on COVID-19 vaccine resistance also verified that those who received information about the safety/efficacy of the vaccine were more likely to take the vaccine [13]. Although some studies examined media representations of vaccine safety and efficacy in the US [14], Australia [15], and Hong Kong [16], few studies gave a comparative study of the representations of the safety and efficacy of COVID-19 vaccines in different socio-political contexts to explicate the dynamic relations between COVID-19 vaccines, media and politics. This is a pity, considering the large number of studies on media representations of COVID-19 in different socio-political contexts [17–20].

This study combines the theories and methods of corpus linguistics (CL) and critical discourse analysis (CDA) to give a corpus-assisted discourse study (CADS) of the representations of COVID-19 vaccines in three representative newspapers from the US, Hong Kong and the Chinese mainland [21, 22]. Particular attention is paid to their preferential ways of representing the safety and efficacy of COVID-19 vaccines at the early stage of vaccine development and distribution [14]. It aims to address two research questions: (1) How do the three newspapers vary in their representations of the safety and efficacy of COVID-19 vaccines? (2) What are the socio-political factors behind their representations of the safety and efficacy of COVID-19 vaccines? The following sections start with a brief introduction to the intertwinement of COVID-19 vaccines and politics. Then we further elaborate the methodology before presenting the findings. The whole article concludes with a brief summary and discussion of the findings.

## 2. Contextualization of this study: COVID-19 vaccines and politics

The development and distribution of COVID-19 vaccines have been severely plagued by geopolitics and nationalism, since a successful vaccine can help a country rebuild their economy and health system and gain advantages over other countries [23]. COVID-19 vaccine research and development have become a new site for global competition and contestation [24] and “a race against time and each other in an unequal world” [25]. This has given rise to “vaccine nationalism”, which usually refers to “the pursuit of vaccines in the national interest, for example, through supply agreements or export bans, including where this might be to the detriment of other countries” [26]. It is in contrast to “vaccine globalism/internationalism”, which underlines global and coordinated access to vaccines [26, 27].

The geopolitics of “vaccine nationalism” has been further complicated by the increasingly conflictual Sino-US relations. Viewing China as a major competitor and challenge to its economy, the US has changed its policy on China since Donald Trump became the US President. This can be seen from the escalating trade war before the pandemic [28]. The intensified geopolitical tensions dampened the potential joint efforts of the two superpowers in vaccine research and development, even though the collaboration between them can contribute to the early recovery of the world from the pandemic [25].

Pursuing its “America First” policy in vaccine development, the Trump administration facilitated and accelerated COVID-19 vaccine development, manufacturing and distribution in the US through its Operation Warp Speed (OWS) [25]. It took a nationalist approach by underlining the early distribution of vaccines in the US [26]. Even though the WHO-backed global COVID-19 vaccine initiative COVAX was established to ensure equitable access to COVID-19 vaccines in June 2020. The Trump administration refused to join COVAX. While the Biden administration promised to join in February 2021, it did not release further details about it and only promised to donate surplus vaccines in the US to countries in need. The global inequality in access to COVID-19 vaccines has been regarded by the WHO’s Director-General, Tedros Adhanom Ghebreyesus, as “catastrophic moral failure” [29].

By contrast, China viewed the development and distribution of COVID-19 vaccines as both a solution to the pandemic and an opportunity to promote its soft power and boost its international image [23]. The Chinese government also offered land, loans and subsidies for vaccine companies to make vaccines along with fast-tracking approvals [23]. Unlike the US, China prefers to take a globalist approach to maximize diplomatic gains of COVID-19 vaccines. Chinese President Xi Jinping promised to make China-made COVID-19 vaccines a public good to ensure global access to COVID-19 vaccines in May 2020. China joined COVAX in October 2020, and launched its “initiative for Belt and Road Partnership on COVID-19 vaccines Cooperation” with 28 other countries in June 2021 [30]. Nevertheless, public trust in Chinese vaccines tended to be affected by the traditional approach (inactivated rather than mRNA) adopted in China’s vaccine development and a series of vaccine safety scandals and incidents in the Chinese mainland during the last decade [31].

Vaccine hesitancy in Hong Kong is both a political issue and a scientific issue. As a Special Administrative Region of the People’s Republic of China, Hong Kong did not manufacture COVID-19 vaccines by itself. The Covid-19 pandemic came as months of anti-extradition bill protests in 2019 gradually cooled down, but tensions continued in Hong Kong. While vaccination rollout was launched on 26 February 2021 in Hong Kong, it lagged far behind other developed countries and the Chinese mainland. Similar to its history as a Chinese city with a colonial past, Hong Kong has COVID-19 vaccine options from the East (i.e., Sinovac) and West (i.e., Pfizer-BioNTech and AstraZeneca) [32]. Although both Pfizer-BioNTech (2 million doses) and Sinovac (2 million doses) were available for Hong Kong people to choose in the first round, many people in Hong Kong were reluctant to take the jab. Apart from the safety and efficacy of the vaccines, the scepticism apparently stemmed from an ingrained distrust in authorities—both the Hong Kong and Chinese governments [33]. Even the choice of the two vaccines (i.e., Sinovac vs. Pfizer-BioNTech) has become a sign of political alignment in Hong Kong. A survey by the University of Hong Kong found that fewer than 30% of people said they would accept the Sinovac vaccine [32].

## 3. Methodology

### 3.1 Data collection and corpus building

This study collects all the news reports concerning COVID-19 vaccines in three newspapers, *China Daily* (CD), the *New York Times* (NYT), and the *South China Morning Post* (SCMP). Our choice of these quality newspapers is “supported by a resumption of public attention in legacy media as reliable sources of information in the COVID-19 pandemic” [34]. Besides, the choice of English-language newspapers results from their functions to communicate competing voices in the international arena. The three newspapers are selected for their functions and influences in their respective socio-political contexts. The NYT is an American daily newspaper. Known as a national “newspaper of record”, the NYT is distinguished for its worldwide readership and international influences in agenda setting [35]. CD is the largest official English-language newspaper in the Chinese mainland. Known as a party organ, it shoulders the responsibility of communicating the voices of the Chinese government to the world [36]. Founded in 1903, SCMP is regarded as relatively the most credible newspaper in Hong Kong [37]. Known for its elite readership and relatively liberal stance, it is also viewed as a “newspaper of record” in Hong Kong [38].

All these newspapers were extracted from the electronic database *Factiva* with the keyword search of *vaccine*. The time span for data collection was from 1 January 2020 to 30 September 2021. Data collection started from 1 January 2020 because COVID-19 did not emerge as a topic before 2020, and ended in September 2021 because vaccine hesitancy was prevalent in the early period of vaccination rollout. All news texts with the keyword were extracted from the electronic database and a research assistant was required to go through these news reports and keep only those news texts which focus exclusively on COVID-19 vaccines. All these news texts were further cleaned by eliminating the irrelevant information in the news texts, such as copyright information, the title of the newspaper, and the date of publication. They were further built into three corpora: the CD corpus (336,799 tokens), the NYT corpus (2,533,114 tokens), and the SCMP corpus (1,004,638 tokens).

### 3.2 Analytic methods and procedure

A corpus-assisted discourse study (CADS) approach features the emphasis on the “balanced synergy” of the methods and theories of corpus linguistics (CL) and (critical) discourse analysis ([C]DA) [21, 22]. On the one hand, it underlines the use of computer-assisted analytic tools to present an efficient analysis of a large sample of data and identify distinct language patterns which cannot be acquired through mere qualitative analysis of a small sample of texts [39]. On the other hand, it highlights the employment of linguistic theories and insights to identify the most prominent and valuable language patterns and present a close analysis of those language patterns in their specific contexts of use and a proper interpretation and explanation of the findings in a certain socio-political context [39]. Therefore, CL can benefit CDA by providing the efficient analytic methods and tools, identifying some prominent language patterns, and providing empirical and scientific evidences for some linguistic insights, and capturing the “incremental” effects of discourse [40]. Meanwhile, (C)DA can benefit CL by providing the required theories and methods to describe and interpret the data, identifying the entry points for further analysis, and revealing the socio-political factors behind the systematic choice of language [41]. This study combines the corpus-analytic methods (i.e., co-occurrence network and collocation) with the discourse-historical approach (DHA) of CDA [42]. The DHA underlines the examination of discourse in its socio-historical context and proposes to analyze texts at three levels: (1) topics/themes, (2) discourse strategies, and (3) linguistic means and realizations [42]. It is critical in that it aims to make apparent the contradictions and inconsistencies in texts and “justify why certain interpretations and readings of discursive events seem more valid then others” [42].

The free text-mining software KH-Coder [43, 44] was used to analyze the topics/themes. KH-Coder uses “Stanford POS Tagger to extract words from English data, R for statistical analysis, and MySQL to organize and retrieve the data” [43]. Higuchi [43, 44] has illustrated how to use this software for quantitative content analysis. KH-Coder was first used to perform a co-occurrence network analysis of the typical tokens used for the safety and efficacy of COVID-19 vaccines. An examination of the word lists of the three corpora generated by Wordsmith 7.0 finds that two prominent tokens can be identified for both safety (i.e., *safe* and *safety*) and efficacy (i.e., *effective* and *efficacy*). Table 1 shows the raw frequencies of the four tokens in the three corpora and their log-likelihood values when the three corpora are compared in turn with a general reference corpus, i.e., the 5 million sampler corpus of the British National Corpus (BNC).

**Table 1:** The occurrences of four related tokens in the three corpora.

	CD		NYT		SCMP	
Tokens	Freq.	LL	Freq.	LL	Freq.	LL
*effective*	355	746.63	1832	1657.68	854	1227.7
*efficacy*	235	1206.42	866	1744.14	980	3319.67
*safe*	272	810	1188	1436.59	475	843.66
*safety*	252	524.56	1446	1390.44	600	847.09

**Fig 1:**
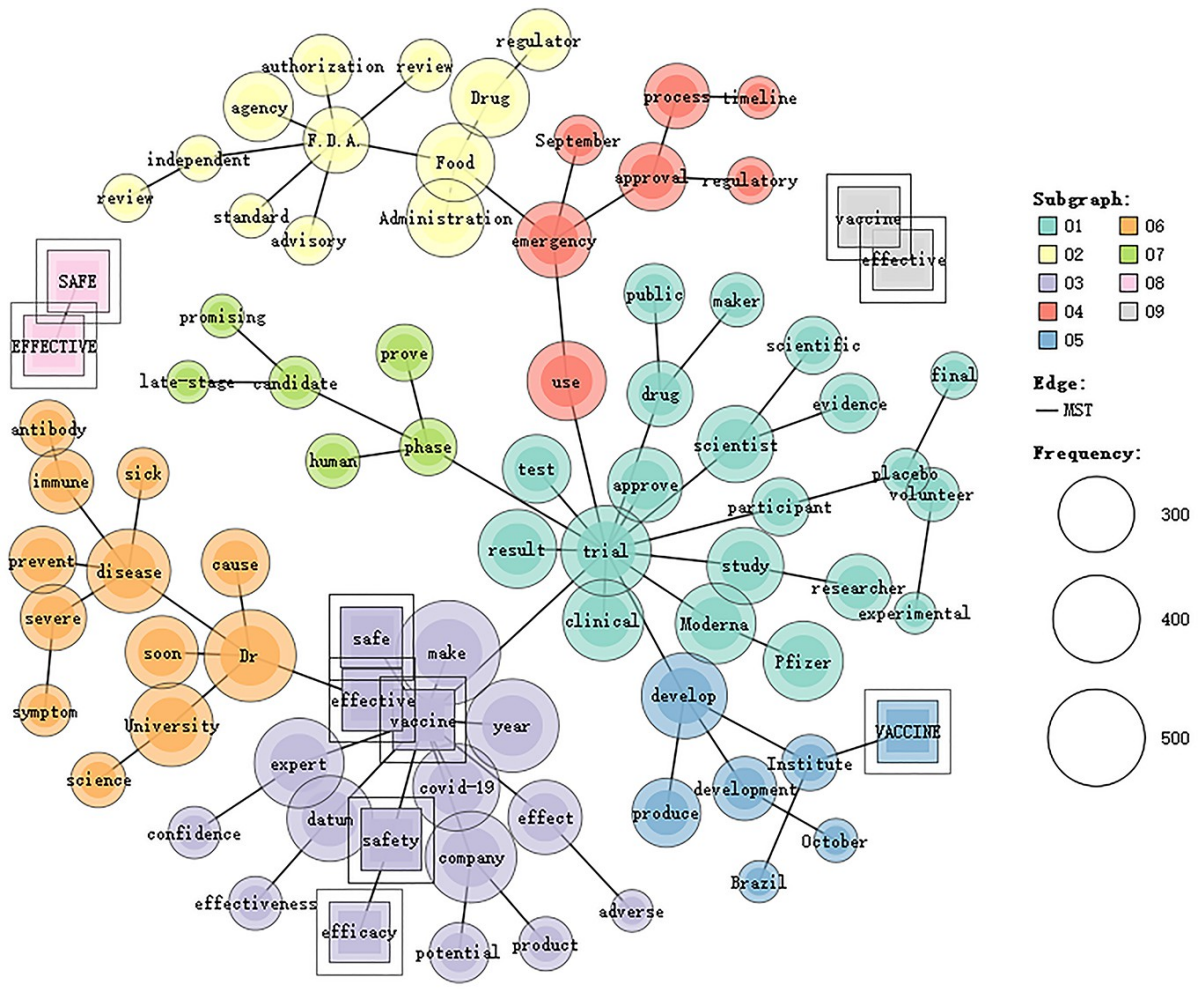
Co-occurrence network of the five tokens in NYT.

Co-occurrence network analysis is a commonly used technique for quantitative content analysis, and the strength of co-occurrence is calculated by Jaccard coefficient which is a similarity measurement of sample sets (i.e. degree of co-occurrence) [45]. In a co-occurrence network, relations of closely connected word nodes are linked by edges (lines), and the different colors represent different clusters of words (see Fig 1). KH coder’s coding option uses a dictionary-based approach and can extract concepts through a pre-specified coding or condition. It can estimate the relations between codes, identify a list of words closely connected with the specified code or search for a document satisfying the specific code [43, 44]. For the current research, a coding rule is defined in a text document which contains two codes safe* (including *safe* and *safety*) and effective* (including *efficacy* and *effective*). To ensure the extracted tokens are directly related to the COVID-19 vaccine, a “direct” code with the token *vaccine* is specified in the search entry. The co-occurrence network analysis can help to identify the most prominent topics and themes surrounding the investigated tokens.

Besides, the Wordsmith Tools 7.0 will be used to identify the distinctive discursive strategies for constructing the safety and efficacy of COVID-19 vaccines by a close analysis of the collocates and their concordance lines. Collocation refers to the “frequent co-occurrence of words within a pre-determined span, usually five words on either side of the word under investigation (node) [46]” [22]. This study focuses on the strongest collocates collocating with the investigated tokens like *effective*, *safe* and *safety* to examine the discursive strategies involved in the construction of the safety and efficacy of COVID-19 vaccines. The strength of collocation is measured in terms of their Log-likelihood values in this study [40]. Particular attention is paid to three discursive strategies: (1) nomination; (2) predication, and (3) intensification/mitigation [47]. The discursive strategies of perspectivation and argumentation are not examined, because they are concerned about the incorporation of different perspectives and the topos used for the justification of a particular stance [47]. Due to the limitation of space, this study addresses only the accumulative stance of each newspaper towards COVID-19 vaccines rather than the different perspectives incorporated and the topos used for the justification of a particular stance [40]. Their preferential ways of constructing the safety and efficacy of COVID-19 vaccines are further interpreted in terms of their underlying ideologies and the politics of globalism and nationalism behind their particular ways of representations [48]. It is expected that a CADS approach can reveal the dynamics between vaccines, media and politics in press representations of COVID-19 vaccines.

## 4. Findings

### 4.1 Analysis of key topics and themes

Fig 1 shows the co-occurrence network of the five tokens (i.e., *effective*, *efficacy*, *safe*, *safety* and *vaccine*) in NYT. These co-occurring tokens are clustered into 9 categories. Category 1 focuses on the clinical trial and test of the two vaccines produced in the US, namely, Moderna and Pfizer. Category 2 concerns the authorization and review of the vaccines by concerned government institutions. Category 3 addresses the safety, efficacy and impact of COVID-19 vaccines. Category 4 is about the approval of vaccines for emergency use. Category 5 highlights the development and production of COVID-19 vaccines. Category 6 underlines the vaccines’ effects in preventing severe symptoms. Category 7 concerns these vaccines which have been proved as promising vaccines in clinical trials. Category 8 underlines the co-occurrence of *safe* and *effective*, while Category 9 highlights effective vaccines. Therefore, NYT is inclined to take a nationalist approach, because it is concerned primarily about the safety and efficacy of COVID-19 vaccines in the US, including Moderna and Pfizer and the foregrounding of F.D.A. in the review and approval of COVID-19 vaccines.

**Fig 2:**
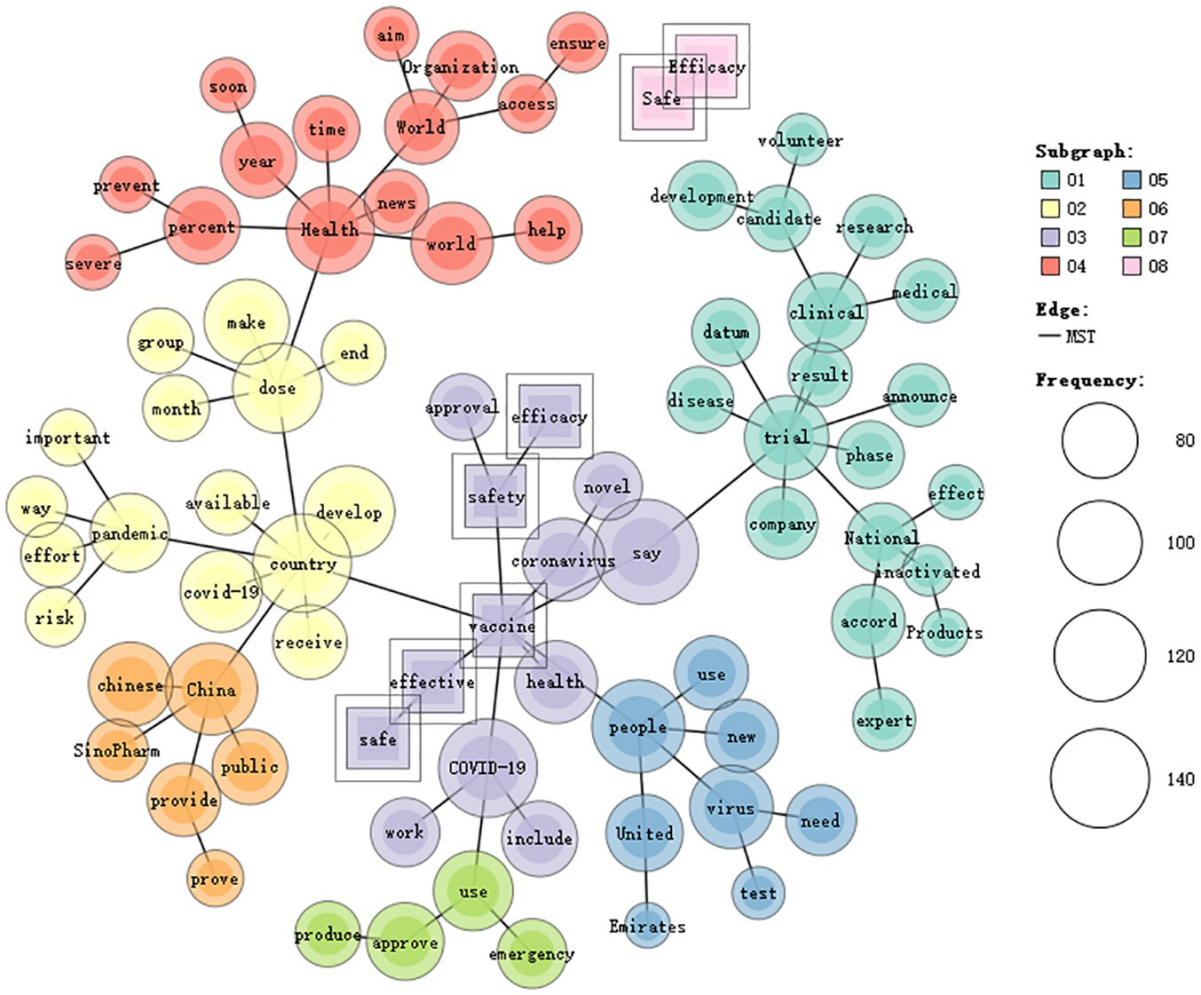
Co-occurrence network of the five keywords in CD.

Fig 2 shows the co-occurrence network of these tokens in CD. These co-occurring tokens are clustered into 8 categories. Category 1 is still about the clinical trial of different candidate vaccines. Category 2 underlines the importance of making vaccines available to different countries. Category 3 highlights the safety and efficacy of COVID-19 vaccines. Category 4 concerns the World Health Organization’s efforts to ensure the world to get access to COVID-19 vaccines in a short time and the COVID-19 vaccines’ impact on preventing severe cases. Category 5 addresses the test of COVID-19 vaccines in United Arab Emirates. Category 6 refers to the providing of Sinopharm vaccine in China. Category 7 is concerned with the approval of vaccines for emergency use. Category 8 underlines the co-occurrence of *safe* and *efficacy* in CD. Therefore, CD tend to take a globalist approach, because it underlines the importance of making vaccines available to different countries. Besides, it prefers to foreground the important role of World Health Organization in ensuring the global access to COVID-19 vaccines.

**Fig 3:**
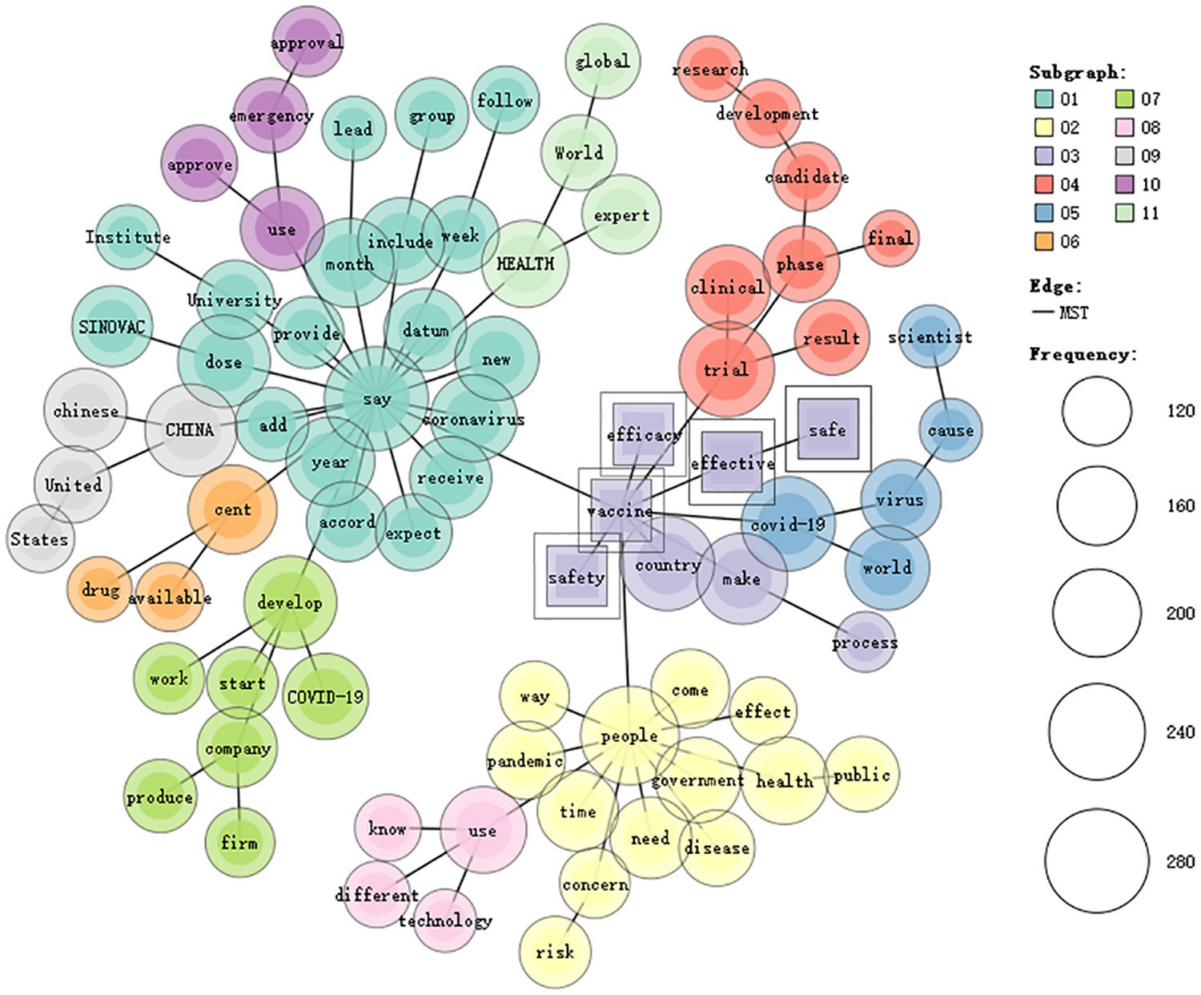
Co-occurrence network of the five keywords in SCMP.

Fig 3 shows the co-occurrence network of these tokens in SCMP. The co-occurring tokens are clustered into 11 categories. Category 1 concerns the speeches about the time and the groups to receive COVID-19 vaccines, in particular Sinovac. Category 2 underlines public concerns about the pandemic and risks. Category 3 focuses on the process to make safe and effective COVID-19 vaccines. Category 4 addresses the results of clinical trials. Category 5 concerns the impact caused by the COVID-19 in the world. Category 6 is about making the vaccines available. Category 7 refers to the development and production of COVID-19 vaccines by companies. Category 8 is about the use of different technologies in producing COVID-19 vaccines. Category 9 highlights the relations between China and the US. Category 10 underlines the approval of COVID-19 vaccines for emergency use. Category 11 suggests the authority of world health experts. Therefore, SCMP underlines public concerns about the pandemic and risks on the one hand and what global health experts say about the production and distribution of COVID-19 vaccines on the other hand. Unlike CD and NYT, SCMP does not foreground the results of clinical trials, which can be attributed to the fact that Hong Kong does not develop vaccines by itself. Therefore, it tends to foreground what global health experts say over the results of clinical trials.

### 4.2 Analysis of the safety of COVID-19 vaccines

The three newspapers’ different ideologies about safety can be revealed through the strategies of **intensification**, **predication**, and **nomination**. Intensification strategy refers to intensifying “the illocutionary and thus the epistemic or deontic status of utterances” [42]. One prominent **intensification** strategy used for the safety of COVID-19 vaccines can be seen from the strong collocation *until* and *safe* in all three corpora. *Until* ranks as the 2^nd^ strongest collocate (30) of *safe* in CD, the 17^th^ (38) in NYT, and the 9^th^ (24) in SCMP. It is used primarily as an intensification strategy to underline when a certain party can be safe. However, in CD, 43% (13) of them are used to underline when each country can be safe (see Example 1), 43% (13) when each person can be safe (see Example 2). Only 7% (2) are used to underline when we can be safe (see Example 3), only 7% (2) when you can be safe (see Example 4). Therefore, CD prefers to take a globalist approach by highlighting the safety of all people and all countries in the world. Examples are as follows:

(1) That the world is divided into the haves and have-nots when it comes to vaccines puts people in all countries at risk, since no country will be *safe* ***until*** all are. (CD, 29/12/2020)
(2) “We need to ensure vaccines for all, because no one is *safe* ***until*** everyone is safe,” she said. (CD, 07/09/2021)
(3) For as UN Secretary-General Antonio Guterres has said, “In an interconnected world, none of us is *safe* ***until*** all of us are safe.” (CD, 25/03/2021)
(4) “It’s really in the self-interest of every country to see everyone vaccinated because you’re not *safe* ***until*** everyone is safe,” she said. (CD, 17/02/2021)

In NYT, only 68% (26) underline when a certain party can be safe, and the rest (32%) highlight when a vaccine can be considered as safe. Besides, instead of highlighting when all countries are safe (3, 12%) (see Example 5), 46% (12) underline when everyone can be safe (see Example 6), 35% (9) when we can be safe (see Example 7), 7% (2) when America can be safe (see Example 8). In other words, NYT is more concerned about the safety of America and Americans rather than people from other countries. Therefore, NYT takes a more nationalist approach than CD in its representations of the safety of COVID-19 vaccines.

(5) But more is needed, especially as scientists make clear that no country is really *safe* ***until*** all are, since continued spread can lead to more variants. (NYT, 19/02/2021)
(6) “I would like to repeat here our urgent warning to all governments: None of us are *safe* ***until*** all of us are safe,” he said. (NYT, 30/11/2020)
(7) “We will not be *safe* ***until*** we can share it with the rest of the world,” said Prime Minister Justin Trudeau of Canada, referring to a vaccine. (NYT, 05/05/2020)
(8) “We know America will never be fully *safe* ***until*** the pandemic that’s raging globally is under control,” Mr. Biden said in a brief appearance at the White House. (NYT, 18/05/2021)

In SCMP, 92% (22) underline when a certain party can be safe. Among them, 82% (18) highlight when one can be safe (see Example 9), 27% (6) when a country can be safe (see Example 10), 14% (3) when we can be safe (see Example 11). It suggests that SCMP is concerned about when everyone and when every country can be safe, even though the former tends to be more emphasized than the latter. This can also be seen from some examples which highlight both everyone and every country at the same time (see Example 12).

(9) Experts have said that no one will be *safe* ***until*** the world is vaccinated. (SCMP, 24/07/2021)
(10) On the question of when China will reopen, he said this month that “no country is *safe* ***until*** all countries are safe”… (SCMP, 24/07/2021)
(11) There is also widespread recognition that none of us is *safe* ***until*** all of us are. (SCMP, 07/05/2021)
(12) “It is in the best interest of all countries to ensure equitable access to new vaccines. No one, no country will be *safe* ***until*** everybody is safe.” (SCMP, 21/09/2020)

Besides, their different ideologies about safety can also be revealed through the **predication** strategies for the token *safe*. Predication strategies refer to “discursive qualification of social actors, objects, phenomena, events, processes, and actions” [42]. While all three corpora underline the safety of COVID-19 vaccines, NYT also foregrounds the safety of its people. This can be seen from the strong verb collocates of *safe* in three corpora. Among top 50 collocates of *safe* in three corpora, lexical verb collocates are as follows:

- **CD**: *proved* (15), *proven* (8), *keep* (7), *feel* (5), *shown* (7), *proves* (3), *found* (6), *ensure* (7)
- **NYT**: *keep* (70), *proved* (27), *shown* (27), *deemed* (16), *proves* (12), *keeping* (17), *feel* (23), *prove* (15), *proven* (11), *ensure* (17)
- **SCMP**: *proven* (15), *prove* (10), *proved* (8), *shows* (7), *ensure* (10), *showed* (10), *shown* (7), *feel* (6), *considered* (6), *indicated* (5)

In three corpora, *safe* shows a strong collocation with those verb collocates which foreground the proof and demonstration of the safety of COVID-19 vaccines, such as *proved*, *proven*, *prove*, *proves*, *showed*, and *shows*. However, *safe* shows the strongest collocation with the verb collocate *keep* (70) in NYT. A close examination of its concordance lines finds that it is used to highlight the aim to keep a certain group of people safe in the US, as in the following:

(13) Without question, the sacrifices required to ***keep*** us *safe* from Covid-19 are costly. (NYT, 12/11/2020)
(14) Do you believe it is important for schools to require vaccinations like these to ***keep*** all students *safe*? (NYT, 15/09/2021)

The same ideology can also be seen from the strong verb collocate *deemed* in the US. It is used to describe some vaccines which are confirmed to be safe by some regulators. It helps to construct the safety of vaccines as a perspective rather than a matter of fact, as in the following:

(15) Though the regulator, the European Medicines Agency, ***deemed*** the vaccine *safe*, the risk of very rare blood clots has led some nations to adapt their approaches. (NYT, 10/06/2021)

Consistent findings can also be found in the **nomination** strategies for the token *safety* in three corpora. Nomination strategies refer to the strategies of labelling social actors, objects, phenomena, events, processes, and actions [42]. They can be revealed through the strong nominal collocates of *safety* in three corpora, as in the following:

- **CD**: *data* (23), *concerns* (13), *standards* (8), *profile* (3), *protocols* (3), *trials* (12), *record* (3)
- **NYT**: *concerns* (102), *data* (158), *issues* (40), *trials* (76), *protocols* (21), *standards* (23), *measures* (32), *scare* (11), *concern* (19), *questions* (22)
- **SCMP**: *concerns* (78), *data* (65), *protocols* (9), *issues* (14), *standards* (11), *profile* (7), *fears* (8), *requirements* (10), *scandals* (5), *concern* (10), *doubts* (6), *confidence* (10)

Among them, all three corpora show a preference for *safety data*, *safety standards*, and *safety protocols*. In a scientific sense, if the data of a vaccine follows the safety protocols and meets the safety standards, it is considered safe. However, all three corpora underline safety concerns. A close examination of their concordance lines reveals that safety concerns are represented in different ways. In CD, they underline safety concerns about foreign vaccines (46%) and no serious safety concerns about Chinese vaccines (54%). In NYT, 25% (26) highlight no safety concerns, 75% (76) safety concerns. In SCMP, only 15% (12) underline no safety concerns, 85% (66) safety concerns. Examples are as follows:

(16) Furthermore, the analysis shows no serious *safety* ***concerns***. (CD, 15/12/2020)
(17) The company said that an independent Data Safety Monitoring Board found no *safety* ***concerns***. (NYT, 28/05/2021)
(18) There are *safety* ***concerns*** with enhancing immunity in patients whose responses are suppressed for a reason. (NYT, 06/08/2021)
(19) In the first 100 individuals, we haven’t encountered *safety* ***concerns*** and these results are encouraging. (SCMP, 31/07/2020)
(20) Those with ***concerns*** over the *safety* of the jabs could consult doctors in face-to-face sessions, Li said. (SCMP, 30/09/2021)

SCMP ranks the first in foregrounding safety concerns, followed in turn by NYT and CD. This can be further confirmed by other negative nominal collocates of *safety* in NYT and SCMP. In NYT, *safety* also shows a strong collocation with some negative nominal collocates, such as *issues* (40), *scare* (11), and *questions* (22).

(21) He noted that the main threat to vaccination programs around the world is misinformation, often related to an unproven *safety scare*. (NYT, 24/11/2020)
(22) During a pandemic, vaccine *safety questions* make headlines. (NYT, 23/03/2021)
(23) Brazil rejected Sputnik V this week over *questions* about its production and *safety*, but the vaccine has been approved for use in dozens of countries. (NYT, 29/04/2021)

Although NYT addresses the negative aspects of the safety of COVID-19 vaccines, it still gives positive presentations of vaccines made in Britain and the US and negative presentations of vaccines made in China and Russia. Even though the vaccine made by AstraZeneca and Oxford shows some safety problems, they are downplayed by such expressions as *no serious safety issues*, *an unproven safety scare*, *and an unsubstantiated safety scare*. Nevertheless, it tends to highlight the unfounded safety questions about vaccines made in Russia and China.

In SCMP, safety also shows a strong collocation with some negative nominal collocates, such as *issues* (14), *fears* (8), *scandals* (5), and *doubts* (6). On the one hand, it highlights that there were no serious safety issues. On the other hand, it prefers to foreground public fears and doubts over vaccine safety and vaccine safety scandals in the Chinese mainland. In other words, it tends to align with the government to show that COVID-19 vaccines do not have serious safety issues, but it also aligns with the public in showing their fears and doubts towards vaccines made in Russia and China. Examples are as follows:

(24) There were *no issues* of *safety*, quality and efficacy, according to officials. (SCMP, 03/04/2021)
(25) Then there are *fears* of vaccine *safety* following reports of side effects and adverse reactions… (SCMP,14/05/2021)
(26) Meanwhile, Russia was the first country to grant regulatory approval for a vaccine, but raised *safety fears* by doing so before large-scale trials were complete. (SCMP, 09/12/2020)
(27) China’s vaccine industry in recent years has been rocked by several *safety scandals*, including substandard vaccines sold for child immunisations. (SCMP, 04/08/2020)

### 4.3 Analysis of the efficacy of COVID-19 vaccines

The different ideologies about the efficacy of COVID-19 vaccines can be revealed through the intensification/mitigation strategies for the token *effective*. As Table 1 shows, *effective* is more frequently used than *efficacy* in both CD and NYT. According to Martin and White [49], meanings can be graded either according to their intensity or amount. An examination of the concordance lines of *effective* finds that the efficacy of COVID-19 vaccines is graded by both **amount** and **intensity**. **Intensifications** within the system of Graduation are, in many instances, realized through the comparative and superlative forms of adjectives [49]. The use of intensification/mitigation strategies can also be manipulative in the representations of the safety and efficacy of COVID-19 vaccines. Examples are as follows:

- **CD**: *most* (24), *highly* (11), *less* (10), *very* (4)
- **NYT**: *highly* (144), *less* (134), *more* (66), *most* (65), *very* (22), *powerfully* (16), *extremely* (14), *remarkably* (12), *particularly* (7), *robustly* (6), *equally* (5), *extraordinarily* (5), *fully* (5), *moderately* (5)
- **SCMP**: *less* (45), *more* (26), *highly* (21), *most* (14), *very* (9)

The majority of these tokens show intensification strategies, while only one token (i.e., *less*) suggests mitigation strategies. In three corpora, *effective* shows a strong collocation with four adverbs, including *most*, *highly*, *less*, and *very*. *Effective* is noted for its collocation with the intensifying superlative *most* (24) in CD, the intensifying adverb *highly* in NYT, and the mitigating comparative *less* in SCMP. An examination of the concordance lines of *most* and *effective* in CD finds that they are used to highlight vaccines as the most effective tool or measures to control the spread of the pandemic in the world. This can also be seen from two other strong collocates of *effective* in CD, i.e., *measures* (16) and *tool* (7). This further confirms that CD prefers to take a globalist approach in its representations of COVID-19 vaccines. Although NYT also foregrounds *most* in its representations of COVID-19 vaccines, it is used mainly to intensify the efficacy of the vaccines (see Example 29).

(28) …that vaccination is a ***most*** *effective* ***means*** to control the spread of the virus and lower the death rate and the severity of the disease should someone become infected. (CD, 19/07/2021)
(29) We are certain we have the best, most tested and ***most*** *effective* vaccine in the world. (NYT, 23/08/2020)

An examination of the concordance lines of *highly* and *effective* in NYT finds that they are used primarily to highlight the high efficacy rate of those vaccines made in the US and Britain. This can also be seen from its preference for other intensifying adverbs for effective, such as *very* (22), *powerfully* (16), *extremely* (14), *remarkably* (12), *particularly* (7), *robustly* (6), *equally* (5), *extraordinarily* (5), *fully* (5), *stunningly* (4), *always* (3), *strikingly* (3). Examples are as follows:

(30) Both vaccines were also ***highly*** *effective* at preventing coronavirus-related hospitalizations in the study from England. (NYT, 01/03/2021)
(31) “…Ninety-nine percent of the experts in this area are convinced these vaccines are ***absolutely*** *safe* in children and adults from what we’ve seen,” she said. (NYT, 12/08/2021)

Although NYT also foregrounds *less* in its representations of COVID-19 vaccines. It underlines that these vaccines are less effective to new variants of the virus in NYT. However, the majority of these statements are mitigated by such expressions as *slightly*, *somewhat*, *seem*, *appeared to*, *indicated*, and *suggested*.

(32) Preliminary studies have shown that some coronavirus vaccines are ***less*** *effective* against that variant. (NYT, 05/02/2021)

However, the collocation of *less* and *effective* in SCMP are used to compare Chinese vaccines with vaccines made in the US and Britain and highlight Chinese vaccines as less effective. This can also be found in the strong collocation of *effective* and *more* in SCMP. They are used to highlight vaccines made in Britain and the US as more effective than Chinese vaccines and the necessity of buying more effective vaccines.

(33) But the Chinese vaccines are far ***less*** *effective* than American ones, he added, leaving an opening for the US. (SCMP, 21/05/2021)
(34) It is also looking at buying different and ***more*** *effective* vaccines such as by Moderna, and plans to order 20 million Pfizer-BioNTech doses to be delivered by the end of the year. (SCMP, 08/08/2021)

The same ideologies about the efficacy of COVID-19 vaccines can also be revealed in the rhetoric of quantification. The rhetoric of **quantification** refers to the use of numerical and non-numerical quantity formulations for argumentative purposes [50]. **Quantification** is a means via which meanings can be graded up or down as force in terms of **Number**, **Mass**, and **Extent** [51]. The rhetoric of **quantification** can be revealed through the numbers immediately before *effective*, which underline the efficacy rate of COVID-19 vaccines. Among the concordance lines of *effective*, 17% (60) in CD, 20% (370) in NYT, and 30% (256) in SCMP have percentage numbers before *effective*. Therefore, SCMP shows the highest preference for percentage numbers, followed in turn by NYT and CD. The most frequent percentage numbers are as follows:

- **CD**: *100* (8), *95* (5), *86* (4), *90* (4), *94*.*5* (3), *91* (3), *70* (3)
- **NYT**: *90* (75), *95* (67), *100* (40), *50* (20), *94* (18), *85* (16), *94*.*5* (11), *79* (10)
- **SCMP**: *100* (28), *90* (27), *50* (23), *95* (20), *78* (19), *70* (13), *79* (13), *87*.*5* (12)

Table 2 shows the distribution of percentage numbers in three corpora. For the percentage numbers below 70, CD featured the lowest preference (7%), while SCMP shows the highest preference (27%). It suggests that CD avoids mentioning the low efficacy rate of COVID-19 vaccines, thus presenting a more positive picture. However, NYT shows the highest preference for the percentage numbers above or equal to 90. This suggests that NYT tends to highlight the high efficacy rate of COVID-19 vaccines. For the percentage numbers above 70 and below 90, CD shows the highest preference, followed in turn by SCMP and NYT. Overall, SCMP presents the most negative picture about the efficacy rate of COVID-19 vaccines by downplaying the high efficacy rate and highlighting the low efficacy rate of COVID-19 vaccines. However, NYT tends to underline the high efficacy rate of COVID-19 vaccines, while CD tends to downplay the low efficacy rate of COVID-19 vaccines.

**Table 2:** Distribution of numbers in three corpora.

	CD		NYT		SCMP	
Types	Freq.	%	Freq.	%	Freq.	%
<70	4	7	60	16	69	27
70–90	23	38	84	23	82	32
> = 90	33	55	226	61	105	41
**Total**	**60**	**100**	**370**	**100**	**256**	**100**

A further examination of these percentage numbers shows the general discursive strategy of “positive self-presentation and negative other-presentation” at work [52]. CD tends to downplay the low efficacy rate of Chinese vaccines (e.g., Sinovac) and highlights the low efficacy rate of Western vaccines. Only one case mentioned the low efficacy rate of 50.65 of a Chinese vaccine Sinovac. However, it presents a complete picture about its efficacy rate for different symptoms, starting from the highest rate (100%) to the lowest rate (50.65%). Besides, it repeatedly highlights that Chinese vaccines are 100 percent effective in preventing severe cases.

(35) Sinovac said that in clinical trials in Brazil involving nearly 12,400 health workers, the vaccine was ***100 percent*** *effective* at preventing COVID-19-related deaths, severe cases and cases requiring hospitalization, 83.7 percent effective for cases showing symptoms and demanding medical treatment, and ***50*.*65 percent*** *effective* for mild cases. (CD, 08/02/2021)
(36) This compares unfavorably with clinical trial data, which found the Pfizer vaccine was ***52 percent*** *effective* after one dose, and 95 percent effective after two. (CD, 21/01/2021)
(37) He added that all approved vaccines are ***nearly* 100 percent** *effective* in preventing severe cases, which is vital to curbing hospitalizations and controlling fresh outbreaks. (CD, 10/04/2021)

For NYT, it underlines both high and low efficacy rates of COVID-19 vaccines. The lowest efficacy rate in NYT is 30 percent. They underline both the low efficacy rate of Chinese vaccines (10, 17%) and the low efficacy rate of Western vaccines against new variants (29, 48%). While underlining the high efficacy rate of American vaccines, it also questions the statement about 100 percent efficacy of Chinese vaccines.

(38) A study in Chile showed that Sinovac was ***only 36 percent*** *effective* in preventing hospitalizations after one shot. (NYT, 27/06/2021)
(39) Pfizer said a vaccine it was developing with BioNTech was found to have been ***more than* 90 percent** *effective* in preventing coronavirus infections, based on a large study. (NYT, 09/11/2020)
(40) No vaccine is ever ***100 percent*** *effective*, experts say… (NYT, 09/06/2021)

SCMP constructs the most negative picture about the low efficacy of COVID-19 vaccines. The lowest efficacy rate is 3 percent in SCMP. The low efficacy numbers are used primarily to refer to the low efficacy rate of Chinese vaccines (50, 72%). Only 7% (5) refer to vaccines from other countries. However, SCMP holds an ambivalent attitude towards the high efficacy rate of Chinese vaccines. 30% (8) underline that no vaccine is 100 percent effective, while 70% (19) underline the vaccine as 100 percent effective.

(41) But the study by the University of Chile also found that one dose of the Sinovac jab was ***only 3 percent*** *effective* against infection, underscoring the need to get fully vaccinated. (SCMP, 10/04/2021)
(42) No vaccine is ***100 percent*** *effective* at stopping the spread of disease or completely safe. (SCMP, 23/02/2021)
(43) The vaccine was also found to be ***100 percent*** *effective* against severe Covid-19 and hospitalization… (SCMP, 23/09/2021)

## 5. Discussion and conclusion

To sum up, CD prefers to take a globalist approach by highlighting the global access to COVID-19 vaccines and the authority of WHO in the global distribution of COVID-19 vaccines [26]. It underlines the interrelations between all people and all countries and highlights COVID-19 vaccines as the most effective means to control the spread of COVID-19. It constructs the most positive picture about the safety and efficacy of COVID-19 vaccines, even though the general strategy of positive representations of the safety and efficacy of Chinese vaccines and negative representations of Western vaccines is still at work in CD. NYT prefers to take a nationalist approach by confining its primary interest to American vaccines. It tends to underline the safety and efficacy of American vaccines and the authority of its own organization (i.e. F.D.A.) in the approval of COVID-19 vaccines. It makes a distinction between the safety of COVID-19 vaccines and the safety of specific groups of people. In other words, even a COVID-19 vaccine has been proved to be safe, it does not mean that it is safe to all groups of people. Therefore, it is less certain about the safety of COVID-19 vaccines by underlining the safety concerns. By contrast, it tends to be most certain about the efficacy of American vaccines. Overall, it still features a positive presentation of the safety and efficacy of Western vaccines and a negative presentation of Chinese and Russian vaccines. Caught between the East and West, SCMP foregrounds both local concerns over the safety and efficacy of COVID-19 vaccines and global authorities’ information about COVID-19 vaccines. It prefers to underline the safety of all people and countries in order to highlight the global access to COVID-19 vaccines. Giving voices to public concerns, it constructs the most negative picture about the safety and efficacy of COVID-19 vaccines. Nevertheless, it still shows its preference for Western vaccines by casting doubts about the safety and efficacy of Chinese vaccines and comparing Chinese vaccines unfavourably to Western vaccines.

Therefore, the global representations of the safety and efficacy of COVID-19 vaccines still tend to be filtered by the prism of local or national concerns [35, 48, 53]. CD aligns with the national interests of the Chinese government in underlining global access to COVID-19 vaccines and the safety and efficacy of Chinese vaccines. It helps to construct a positive image of Chinese COVID-19 vaccines and a responsible image of the Chinese government [23]. However, the globalist discourse has also been constructed in a nationalist manner by praising Chinese COVID-19 vaccines and downplaying Western COVID-19 vaccines [19]. The co-existence of globalism and nationalism in Chinese national English-language newspaper thus contributes to the enhancement of China’s national image and world influence [54]. The NYT also aligns with the US government and Americans in underlining the safety of COVID-19 vaccines and Americans, to the ignorance of the safety of people from other countries. Pursuing its “America first” policy, the Trump and Biden administrations foreground national interests over global interests and refuse to take the global responsibilities for developing and distributing COVID-19 vaccines. Besides, the bloc logic of the Cold War was still at work in NYT’s differential representations of Chinese and Russian vaccines and Western vaccines [55]. With its origin in the west, SCMP shows alignment with public concerns and global authorities and disalignment with Chinese vaccines and the Chinese authorities. This can be attributed to the ingrained distrust of the Chinese government and Chinese vaccines and the continued tension between Hong Kong and the Chinese mainland. Therefore, the representations of COVID-19 vaccines in the NYT, CD and SCMP also feature a high degree of politicization and polarization [20]. As Zhou [25] argues, vaccine nationalism “should not be simplified as a sole product of domestic nationalism or a form of national crisis management strategy” but “a mix of geopolitics, international economic inequalities, global capitalism, and ‘me-first’ politics”.

A corpus-assisted discourse study of the representations of the safety and efficacy of COVID-19 vaccines in the three newspapers, therefore, cannot only reveal their preferential choices at different levels of discourse, but, more importantly, the socio-political factors behind their representations [22]. The findings demonstrate that the safety and efficacy of COVID-19 vaccines in news media are “a constructed reality” [10] and an important site of ideological struggles [56]. Due to the limitation of space, this study only addresses the nomination, predication and intensification/mitigation strategies, future studies will give a close examination of the perspectivation and argumentation strategies to further illuminate the dynamics between vaccine, media and politics. It hopes that it can lead to more studies on media representations of the safety and efficacy of COVID-19 vaccines in other socio-political contexts.
